# Time and cost associated with utilization of services at mobile health clinics among pregnant women

**DOI:** 10.1186/s12913-018-3736-z

**Published:** 2018-12-03

**Authors:** Nyasule Neke, Antonius Reifferscheid, Barbara Buchberger, Jürgen Wasem

**Affiliations:** 1 National Institute for Medical Research, Mwanza Centre, Isamilo Street, P O BOX 1462, Mwanza, Tanzania; 2 Alfried Krupp von Bohlen und Halbach, Institute for Health Care Management and Research, University of Duisburg-Essen, Thea-Leymann-Str. 9, 45127 Essen, Germany

**Keywords:** Mobile health clinic, Antenatal care, Patient cost

## Abstract

**Background:**

Antenatal care (ANC) is provided for free in Tanzania in all public health facilities. Yet surveys suggested that long distances to the facilities limit women from accessing these services. Mobile health clinics (MHC) were introduced to address this problem; however, little is known about the client cost and time associated with utilizing ANC at MHC and whether these costs deter women from using the provided services.

**Methods:**

Client-exit interviews were conducted by interviewing 293 pregnant women who visited the MHC in rural Tanzania. Two subgroups were created, one with women who travelled more than 1.5 h to the MHC, and the other with women who travelled within 1.5 h. For each subgroup we estimated the direct cost in US$ and time in hours for utilizing services and they hinder service utilization. The Wilcoxon–Mann–Whitney rank sum test was performed to compare the differences between the estimated mean values in the two groups.

**Result:**

Total direct cost per visit was: US$2.27 (SD = 0.90) for overall, US$2.29 (SD = 1.03) for those women who travelled less than 1.5 h and US$2.53 (SD = 0.63) for those who travelled more than 1.5 h (*p* = 0.08). Laboratory and medicine cost accounted for 70 and 16% of the total direct cost and were similar across the groups. Total time cost per visit (in hours) was: 3.75 (SD = 1.83), 2.88 (SD = 1.27) for those women who travelled less than 1.5 h and 5.02 (SD = 1.81) for those who travelled more than 1.5 h (*p* < 0.01). The major contributor of time cost was waiting time; 1.89 (SD = 1.29) for overall, 1.68 (SD = 1.02) for those women who travelled less than 1.5 h and 2.17 (SD = 1.57) for those who travelled more than 1.5 h (*p* = 0.07). Participants reported having missed their scheduled visit due to lack of money (15%) and time (9%).

**Conclusion:**

Women receiving nominally free ANC incur considerable time and direct cost, which may result in an unsteady use of maternal care. Improving availability of essential medicine and supplies at health facilities, as well as focusing on efficient utilization of community health workers may reduce these costs.

**Electronic supplementary material:**

The online version of this article (10.1186/s12913-018-3736-z) contains supplementary material, which is available to authorized users.

## Background

Inequality in physical access to ANC is still a problem in Tanzania. Recent evidence indicates that women in urban areas are more than twice as likely as women in rural areas to receive ANC from a skilled health provider [1]. Measures to address physical accessibility of health care are being implemented, and specifically, the Tanzanian Ministry of Health and Social Welfare is deploying MHC throughout the year in order to improve physical access to essential health interventions including ANC [2]. These MHC are reported to have increased access to essential interventions among pregnant women residing in hard to reach areas in the country [3, 4]. Yet, little is known about how much it costs for women to utilize these services at the MHC.

A body of evidence suggests that users’ costs, too, can prevent access to maternal health interventions. Scholars reported a range of hidden costs incurred by women when they utilize different types of maternal health services [5, 6]. These costs may be substantial as observed in a study in Kenya, which suggested that informal fees for maternal health services accounted for 59% of total out-of-pocket expenses paid by pregnant women [7, 8]. Additionally, there are hidden costs associated with transport to a health facility and food costs for the pregnant women. These direct costs are estimated to constitute at least 50% of all direct costs [5, 6]. While the cost of using service at static health facilities can be found in the literature, little has been documented when it comes to the cost of utilization of service offered at the mobile clinics. What this study adds to the existing literature is the comparative information on costs across pregnant women according to the travel duration to the mobile clinics, as well as the taking into account of the time cost of household members taking over their responsibilities during their absence.

The relative cost caused by women when they utilize ANC has significant implications for continuity of care. ANC links the woman and her family with the formal health system, increases the chance of using a skilled attendant at birth, and contributes to good health throughout the life cycle of the baby [9]. Therefore, if ANC is associated with higher patient cost in terms of time or due to out-of-pocket spending as a result of lack of medical supplies, then this may discourage women from remaining in care [10, 11]. To provide insight into the true cost of health care seeking at the MHC, we set out to measure the financial and time-related cost of health care utilization among pregnant women receiving care at these clinics in rural Tanzania. We assessed direct cost and time cost associated both with accessing ANC at the public owned MHC and with using a household helper who assists them or works on their behalf while they attend the clinic.

## Material and methods

### Study setting

The study was carried out in the Kisarawe district in the coast region of Tanzania from November 2015 to June 2016. The district has a population of 101,598 out of which 50,967 are women and 25,779 are of reproductive age as estimated from the 2012 national population census [12]. The district belongs to the lesser developed parts of Tanzania and about 90% of its population lives in rural areas on subsistence farming [13]. The district has a total of 37 governmental dispensaries, eight private dispensaries and four health centres (including one non-governmental centre). The first level of care in the region is represented by dispensaries and health centres. MHC operates in 20 villages, which have been classified as “remote” by using certain criteria (e.g. populations residing more than 5 km from the health facility, or populations residing less than 5 km from the health facility which, however, lacks personnel and/or essential medical equipment would fall into that category) [3, 4].

### Cost methodology

We adopted a patient perspective to ascertain the cost of health care utilization. As the main aim of this study was the estimation of the client’s direct cost and time cost resulting from seeking a free ANC, it was justified to concentrate on service users rather than on doing a household survey. We used a convenient, non-probability sampling technique. Our subjects were enrolled according to their availability and accessibility. This method was selected because it is quick, inexpensive, and convenient. In our situation, the accessible population were pregnant women attending the antenatal clinic at the mobile health clinic in Kisarawe District. Therefore, within the study period, any woman who seek ANC at any mobile health clinic in Kisarawe District and who agreed to participate to the study met the eligibility criteria and were included in this study.

Type, amount, and extent of the cost incurred by women were assessed by conducting key informant interviews (KII) with ten pregnant women who seek services at the mobile health clinic prior to the design of the interview guide. The information gathered from the KII together with information based on literature was used to create the structured questionnaire. The structured interview questionnaire (see Additional file 1) was administered to a total of 293 pregnant women attending the MHC from November 2015 to June 2016. Women signed an informed consent form (see Additional file 1), and all interviews were done in private with only interviewers and respondent being present.

The questions focused on several aspects regarding cost and time spent: Time spent on travelling, waiting and consultation at the MHC, cash payments for services, travel, drugs and supply cost. Interview data on time spent on services was compared and verified by observing waiting and consultation time at the MHC. Information on travel cost was verified by comparing with the public transport rates in the rural areas, while information on the cost of prescribed medicines was compared with the Tanzania Medical Store Department drug cost lists. Based on recommendations from cost guidelines [14, 15], all cost data were collected in Tanzanian shillings and converted to USD for the exchange rate of the year 2016 in which 1US$ was equivalent to 2200 Tanzanian shillings [16].

### Measurements of direct cost incurred

We collected data on expenditures on four broad categories: Cost of visits to the clinic, informal payments paid to health workers, payment due to medicine and laboratory investigations. Expenditures associated with clinic visits were assessed on a per-visit basis. For instance, patients were asked: *“Did you pay to see the health provider today? If yes, how much did you pay? What means of transport did you use to come to the clinic today? If you paid for transport, how much did you pay today?”.* We also asked questions on overnight stays in which women were asked if they had to pay for accommodation to stay the night nearby.

We also measured expenses incurred for medicine and laboratory investigations by asking a question referring to the visit made during the current pregnancy: “*Did you receive all needed medicines and investigations?”.* If the answer was “no”, the follow-up question was asked whether they have to go to the private provider and drug outlet to buy the medicine they were prescribed or to do the investigations which they could not find at the mobile clinic. A similar approach was taken to measure the amount of money they have used for food by asking about the events that happened during their visits during this pregnancy. We also asked about the frequency of these events; although and because our unit of estimation was per visit, we did not take into account the frequency of these payments.

### Measurement of time cost

Data were also collected on the time-related cost associated with clinic visits. Data were collected on time (in hours) spent travelling to the clinic, and time spent at the clinic by asking questions like: *“How long did it take to travel from your home to this clinic? How long in total does it take for you to finish all that you need here at the clinic, from seeing the health provider and taking the needed medication to investigations?”.* Total time cost accounted only for waiting time, consultation time and travel time for a one-way journey. Travel cost was estimated as one-way because interviews revealed that women utilized the time and costs while travelling back to their houses by going to the market or to attend other social activities, hence it seems appropriate to account only for the travel cost and time for only one way.

### Time and cost as a barrier to service utilization

Patients were asked whether time and cost prevented them from utilizing health care, using questions like: *“In the last 2 months, have you ever missed your scheduled visit due to lack of money? If yes, how often?”* and *“In the last 2 months, have you ever missed your scheduled visit due to lack of time? If yes, how often?”.* Similar questions were asked for unscheduled visits. We constructed a dichotomous variable “cost as a barrier”, which took the value of 1 if individuals reported missing either their scheduled or unscheduled visit due to lack of money, or 0 for those who answered no. We also generated a dichotomous variable of “time as a barrier” which took the value of 1 if individuals reported missing either their scheduled or unscheduled visit because of time, or 0 for those who answered no.

### Data cleaning, data entry and statistical analysis

A double data entry was done using EpiData software version 3.1. Data were cleaned and extracted in STATA version 12. Data were grouped into women who travelled for more than 1.5 h and the ones who travelled less than 1.5 h to allow comparison of the mean time utilized, mean cost and mean time cost between the groups. The cut point of 1.5 h was based on recent findings on a study done in Tanzania that modelled the geographic access of emergency obstetrics and neonatal care [17]. In the study, it was observed that only 13% of women can reach health care facilities within 2 h on foot and almost 32% of live births were among women residing in areas where it is impossible to reach facilities within 2 h [17]. That indicated that the World Health Organisation(WHO) [18] optimal travel distance of 2 h is in fact not perceived as “optimal” for the majority, especially in rural and remote settings like in Tanzania. Based on that information and the information on the mode of transport of our participants, 1.5 h were taken as a compelling cut point for travel time.

The demographic summary statistics such as proportions of women belonging to similar occupations, education levels, and parity levels and their percentages were computed. Summary statistics on cost and time such as mean and their standard deviation were also computed and compared by the group. The Wilcoxon–Mann–Whitney rank sum test was performed to compare the differences between the estimated mean values in the two groups because the data were positively skewed. A *p*-value of less than 0.05 was considered statistically significant. However, and because no authoritative reference for setting the significance level exists [19–22], we also reported the real *p*-values and standard deviations of our estimates. Unlike in other medical research, presenting the original data in cost analysis and their distribution is argued to give the reader a more accurate understanding of the similarities and differences in cost than focusing on *p* values alone [14, 15, 23, 24].

## Results

### Demographic and obstetric characteristics of participants

Table 1 below presents the demographic characteristics of the participants by the distance from their household to the point of care. The mean age of women who participated in this interview was 25(SD = 6.66) years. Almost 26 and 22% were primigravidae and secundigravidae, respectively, with the rest of the women being multigravida. The majority of these women were officially married (76%), while few were either cohabiting (19%) or single (5%). A large number had no formal education (70%) and they were working in informal sectors like farming and livestock keeping (35%), food vending (28%), livestock keeping alone (16%) and 7% were engaged in farming only. About 11% were housewives and women who were not able to work due to illnesses accounted for about 0.34% while only 2% were employed in the formal sector.

**Table 1:** Participants’ demographic and obstetric profiles by residence from the point of care

		Total participants =293	≤ 1.5 h travel	> 1.5 h travel	*p* value
			*n* = 173 observation	*n* = 120 observations	
		n (%)	n (%)	n (%)	
Marital status	Cohabiting	55(18.77)	50(28.90)	5(4.17)	
	Married	222(75.77)	118(68.21)	104(86.67)	
	Single	16(5.46)	5(2.89)	11(9.17)	< 0.001
Marriage type	Monogamous	135(40.74)	101(64.24)	34(30.16)	
	Polygamous	142(51.26)	67(35.76)	75(69.84)	< 0.001
Level of education	No formal education	206(70.31)	97(56.07)	109(90.83)	
	Primary education	80(27.30)	69(39.88)	11(9.17)	
	Secondary education	7(2.39)	7(4.05)	0(0.00)	< 0.001
Occupation	Cannot work	1(0.34)	0(0.00)	1(0.83)	
	Farming &livestock keeping	103(35.15)	55(31.79)	48(40.00)	
	Farming only	21(7.17)	21(12.14)	0(0.00)	
	Food vendor	83(28.33)	53(30.36)	30(25.00)	
	Formally employed	6(2.05)	4(2.31)	2(1.67)	
	Housewife	31(10.58)	19(10.98)	12(10.00)	
	Livestock keeping only	48(16.38)	21(12.14)	27(22.50)	0.001
Religion	Christian	90(30.72)	37(21.39)	53(44.17)	
	Muslim	143(48.81)	101(58.38)	42(35.00)	
	Pagan	60(20.48)	35(20.23)	25(20.83)	< 0.001
Parity	Zero Parity	76(25.94)	47(27.17)	29(24.17)	
	Para One	64(21.84)	40(23.12)	24(20.00)	
	Multiparity^a^	116(39.59)	72(41.62)	44(36.67)	
	Grandmultiparity^b^	37(12.63)	14(8.09)	23(19.17)	0.049
Timing of ANC booking	First trimester	256(87.37)	143(82.66)	113(94.17)	
	Second trimester	37(12.63)	30(17.34)	7(5.83)	0.004
Place of first antenatal clinic	Static health facility	96(32.76)	70(40.46)	26(21.67)	
	Mobile health clinic	197(67.24)	103(59.54)	94(78.33)	0.001
Place of delivery of the previous child^c^	Health facility	109(43.25)	64(46.04)	45(39.82)	
	Home	143(56.75)	75(53.96)	68(60.18)	0.322
Mode of transport	Own Bicycle	16(5.46)	5(2.89)	11(9.17)	
	Public Transport	5(1.71)	5(2.89)	0(0.00)	
	Rented Motorcycle	2(0.68)	1(0.58)	1(0.83)	
	Own Motorcycle	33(11.26)	22(12.72)	11(9.17)	
	Walking	231(78.84)	137(79.19)	94(78.33)	
	Others	6(2.05)	3(1.73)	3(2.50)	0.086
Household helper	Friend	5(1.71)	5(2.89)	0(0.00)	
	Immediate family member	278(94.88)	158(91.33)	120(100.00)	
	Nobody	10(3.41)	10(5.78)	0(0.00)	0.004
Direct payment to household helper	Yes	2(0.69)	2(1.19)	0(0.00)	
	No	286(99.31)	166(98.81)	120(100.00)	0.230
Direct payment for transport	Yes	45(15.36)	27(15.61)	18(15.00)	
	No	248(84.64)	146(84.39)	102(85.00)	0.887

Note:

*N* denotes total number, *n* denotes number in the subgroup

^a^ = 2–4 previous pregnancies; ^b^ = 5 and above previous pregnancies; ^c^ = excludes zero parity women

*p* value based on Pearson chi-square

The groups differed in terms of educational level, type of marriage, occupation and marital status. The majority of women residing more than 1.5 h away from the location of the mobile health clinic were more likely to be involved in both farming and livestock keeping, they were in a polygamous type of marriage and had no formal education. Apart from the differences observed for the place of delivery, payment to household helpers and actual payment for transport, other observed differences in the client characteristics presented in Table 1 were significant based on Chi-square *p*-value.

### Time and cost associated with utilizing care at the mobile health clinic

Table 2 presents the estimated time, direct cost and indirect cost associated with utilizing ANC at the mobile health clinic. On average, women spent 3.75 h (SD = 1.83) for a visit, in which those who travelled more than 1.5 h spent 5.02 h (SD = 1.81) and those who travelled less than 1.5 h were estimated to incur a time cost per visit of 2.88 h (SD = 1.27). The overall estimated waiting, travel and consultation times were 1.89 (SD = 1.29), 1.39 (SD = 1.00) and 0.48 (SD = 0.10) hours, respectively. The main drivers of total time cost were waiting time and travel time. All of these parameters differed depending on the participant’s distance of travel from the mobile health clinic.

**Table 2:** Time cost, direct and indirect cost of utilizing ANC at the mobile clinic by client residence from the point of care

	Total participants				≤1.5 h travel				> 1.5 h travel				*p* value
	Number of observations = 293				Number of observations = 173				Number of observations = 120				
	Mean	SD	Min	Max	Mean	SD	Min	Max	Mean	SD	Min	Max	
I: Time spent in hours													
Consultation	0.48	0.10	0.25	0.75	0.49	0.11	0.25	0.75	0.45	0.08	0.25	0.50	0.0312
Waiting time	1.89	1.29	0.05	6.00	1.68	1.02	0.05	4.50	2.17	1.57	0.83	6.00	0.0730
Travel time	1.39	1.00	0.02	4.25	0.70	0.47	0.02	1.50	2.39	0.66	1.75	4.25	< 0.001
Household helper time	4.16	2.45	4.99	10.12	2.75	1.77	0.50	8.62	6.20	1.75	3.02	10.12	< 0.001
Total time spent	3.75	1.83	0.66	8.99	2.88	1.27	0.67	6.75	5.02	1.81	2.50	8.99	< 0.001
II. Cost incurred in US$													
Food cost	0.19	0.26	0.00	0.91	0.11	0.18	0.00	0.77	0.31	0.32	0.00	0.91	< 0.001
Laboratory test cost	1.69	0.82	0.00	3.40	1.71	0.91	0.00	3.40	1.69	0.68	0.00	2.72	0.7426
Medicine cost	0.39	0.23	0.00	0.59	0.41	0.21	0.00	0.68	0.36	0.26	0.00	0.59	0.7438
Travel cost (one way)	0.35	0.88	0.00	3.64	0.36	0.86	0.00	3.18	0.34	0.91	0.00	3.64	0.8511
Household helper cost	0.02	0.28	0.00	3.64	0.04	0.37	0.00	3.64	0.00	0.00	0.00	0.00	0.2381
Total cost in US$	2.27	0.90	0.00	3.40	2.29	1.03	0.01	4.36	2.53	0.63	1.36	3.86	0.0829

Note:

*p* value based on Wilcoxon–Mann–Whitney rank sum test

Travel cost and household helper cost were excluded in the total cost calculations because only 2 and 45 participants, respectively, reported to directly pay for transport and household helper

Overall, total direct cost per visit was: US$2.27 (SD = 0.90) for overall, US$2.29 (SD = 1.03) for those women who travelled less than 1.5 h to reach the mobile health clinic and US$2.53 (SD = 0.63) for those who travelled more than 1.5 h to the mobile health clinic (*p* = 0.08). The cost incurred due to prescribed laboratory investigation was the main driver of the total cost accounting for more than 70%. 45 women incurred the cost of transport in the amount of US$0.34(SD = 0.88) for one-way travel. No relevant cost for household helpers was reported because only two participants (all from the group that travelled less than 1.5 h) paid their household helper and they incurred US$0.02(SD = 0.28) per visit. 45 participants reported actual spending on transport and they paid on average US$0.37 for one-way travel.

### Time and cost as barriers to ANC utilization

As presented in Table 3 below, the majority of women in this study indicated that neither time (91%) nor cost (85%) is a barrier to service utilization. Out of the 26 women who reported that they had missed their scheduled visit due to lack of time, 58% reside within a limit of 1.5 h travel to the point of care. On the other hand, 57% of the 44 women who reported to have missed their scheduled visit due to cost reside more than 1.5 h travel from the point of care. None of the women reported missing their unscheduled emergency visit due to lack of time, but 49 participants (17%) indicated that they had missed their unscheduled visit once, due to lack of money during this pregnancy. The majority of those who reported not going to the next health facility when they felt they needed an emergency care due to cost were those who travelled more than 1.5 h to reach the mobile clinic.

**Table 3:** Time and cost as barrier to service utilization

			Travel time ≤ 1.5 h	Travel time > 1. 5 h	*p* value
		*N* = 293	*n* = 173 obs.	*n* = 120 obs.	
		n (%)	n (%)	n (%)	
I: Time as barrier					
Scheduled visit	No	267(91)	158(91.33)	109(90.83)	
	Yes	26(9)	15(8.67)	11(9.16)	0.883
If yes, how often	Once	15(58)	4(26.67)	11(100)	
	Twice	7(27)	7(46.67)	0(0)	
	More than twice	4(15)	4(26.67)	0(0)	0.001
Unscheduled visit	No	293	173(100)	120(100)	
	Yes	0	0	0	
II: Cost as barrier					
Scheduled visit	No	249(85)	154(89.02)	95(79.17)	
	Yes	44(15)	19(10.98)	25(20.83)	0.020
If yes, how often	Once	14(32)	6(31.58)	8(32.00)	
	Twice	19(43)	11(57.89)	8(32.00)	
	More than twice	11(25)	2(10.53)	9(36.00)	0.107
Unscheduled visit	No	244(83)	155(89.60)	89(74.17)	
	Yes	49(17)	18(10.42)	31(25.83)	0.001
If yes, how often	Once	22(45)	6(33.33)	16(51.61)	
	Twice	27(55)	12(66.67)	15(48.39)	0.215

Note: *p* value based on Pearson chi2 test

## Discussion

This study assessed the direct and indirect cost as well as time incurred by pregnant women when they utilize ANC at the public owned MHC. The estimated total cost and time cost were US$2.27 and 3.75 h, respectively, and the time cost for a household helper was 4.16 h. All of these estimates varied according to the time that a woman had travelled to the mobile clinic. The results not only provided evidence that free services are not actually free due to substantial direct cost (for prescription and laboratory), but the data also suggested that waiting time is a large contributor to time cost. To some extent, our results expanded on those reported previously in Tanzania [25] by documenting direct cost and time cost as well as time cost incurred by caretakers who help these women during their absence when they seek health care at the MHC.

Most mothers in Tanzania, as in other countries, are very busy and work on average for 55.9 h a week [26]. The time that they take to access health care draws on their time and can add to the burden that they already have. Our study indicates that women spend on average 3.75 h when they utilize ANC. This time was largely driven by long waiting time, which accounted for about 50% of total time spent. Our estimates are almost twice as high as those reported in a study conducted in Tanzania suggesting that women spend 70 min (1.17 h) when utilizing ANC at the dispensaries in Tanzania [25]. In both studies, waiting hours contributed half of the total time spent. Long waiting time is documented to affect health care and treatment in crucial aspects of care like adherence to care and treatment [27–29].

On average, women travel 1.39 h to reach the mobile clinic. No study was identified that had estimated client travel duration when they use services at the MHC. Nevertheless, our estimates are higher than those reported by other studies that investigated clients’ costs at the static health clinics. A study done in the southern part of Tanzania indicated that women travel on average for 30 min to reach the dispensary [25]. Travel duration is among the factors that hinder continuity in maternal health care utilization as suggested in previous studies exploring ways to improve uptake of childbirth services in rural areas in Tanzania [12, 30, 31]. On the other hand, travel costs were slightly higher among those who travelled for 1.5 h as compared to their counterpart. This is not surprising because most of the participants who travelled for less than 1.5 h came to the clinic by foot. However, these findings should be interpreted with caution because the estimates for travel cost came from a very small sample size (45 participants) and that may be not sufficient to draw plausible conclusions [32].

The extent to which time as a resource is needed for a woman to utilize health care also includes household members. Noted in this study was that women require help from household members when they seek care and the time that these relatives invest on a woman’s visitation should not be ignored. Women rely heavily on their social networks when they seek health care as suggested in this study. These findings are supported by theories of social capital [33] and social disorganization [34, 35]. These theories provided a framework for understanding the mechanisms through which communities and families may influence the utilization of health services. Studies in sub-Saharan Africa had indicated that availability of social support might enable pregnant women of the same community or neighbourhood to turn to each other when they need a favor, such as a childcare during prenatal visits [36–39]. Studies have also recommended the need to recognize the lack of social support as among the barriers to health care [36, 40]. They also indicated that there are trade-offs made by women before they leave their home to seek maternal health care.

In Tanzania, ANC as part of maternal health services is provided free of charge in all public owned health outlets [41]. Yet our findings suggest that financial protection from such cost is not common practice. Private expenditure on medication and laboratory investigation are among the main contributors to direct patient cost accounting for US$2.08 (87% of the direct cost). The obvious reason for these out-of-pocket expenditures on drugs and investigation in this setting is the frequent lack of drugs in public health facilities. Our findings indicated in this study that almost 85 and 94% of women reported to have missed their needed medication and investigation at the mobile health clinic due to medicines that have run out of stock and lack of reagents, respectively. Lack of medicine and reagents is a common problem in Tanzania as reported by several studies and jeopardizes the quality of maternal health care [10, 11, 28]. A recent survey in Tanzania indicated that this chronic problem does not only affect patient care and health outcomes but also extends to affect staff morale and increases workloads [42]. The situation is similar in other parts of sub-Saharan Africa, in spite of efforts that are made by countries to strengthen the provision of pharmaceuticals [43].

Although food cost did not appear to contribute largely to the direct cost, it is worth to comment how it varied between those respondents who travelled more than 1.5 h to reach the mobile clinic and their counterparts. This was not a surprising finding since women who have to travel for a long time would ideally incur the cost of food. Our findings resonate similarly to those estimated by Ngalesoni and her colleagues where they reported significant food cost especially to those clients who resided in rural districts like ours [44].

The current gross domestic product per capita for the Pwani region in which the Kisarawe district is located is US$470 [13], implying that women in this study spend on average 0.5% of their annual income on utilizing ANC services per visit. These estimates are lower than the health expenditures per capita for the Tanzanian as a general population which is 5% of the total expenditures per capita [45]. However, for a woman to spend 0.5% of their annual income per capita for one ANC visit is alarming. Such a high financial burden on clients for preventive services patients may affect their health-seeking behavior and hence lead to low utilization of health services as reported elsewhere [8, 46, 47].

Given the significance that time and cost have in health care utilization, it is important to note those women who reported non-utilization of health care due to time and direct cost. In our study, the proportion of those who reported to miss their scheduled visits due to lack of time (9%) and cost (15%) were lower compared with the overall estimates from the national survey which suggested that 42 and 50% of women could not access health care due to lack of money and time [12]. This may suggest that the mobile clinics have to some extent reduced those barriers to accessing care for rural pregnant women. While it was encouraging to observe that lack of time will not prevent women from seeking care in case of emergency, it was interesting to see that this was the case if they do not have money as indicated by 16% of our participants.

Several policy implications stem from these research findings. As already noted, the problem of medicine and reagents being out of stock impedes the intention of the free service policy in maternal health care [10, 42]. Therefore, ensuring that this problem is addressed is essential in order to reduce the hurdles that women have to face when they access essential health care services. Having strategies that can address and alleviate if not reduce this problem will allow women to receive necessary health interventions as per policy. Also, waiting time appears to increase the time cost and might influence uptake of services as seen in other studies [8, 27, 48], meaning policy measures like task shifting which can reduce workload to the providers [48–50], may reduce the time that women have to wait for the mobile health clinic. Nevertheless, there is a considerable danger that women will either not use this service or that they will need to borrow money to cover the expenses associated with health care. There can be no dodging the need to reduce these cost, but the best way of doing this is not obvious [7, 51]. The findings point to the major drawbacks of either the health systems as a whole or weakness in some of its pillars, especially the human resources and availability of essential medicines.

Our study has some limitations. First, due to the nature of the clinic-based sampling strategy, we excluded people in need of ANC but without access, including those who did not access health care because they did not get permission from their spouses. In the recent demographic survey it was found that long travel distance, cost, not wanting to go to the clinic alone, and failure to get permission from spouses to go to the clinic were among the most important barriers to service utilization [12]. Second, it is possible that our cross-sectional comparisons across client groups –according to travel time – were confounded by unobserved factors, as in most observational studies, unmeasured factors could influence our effect estimates. Third, hidden cost related to care seeking outside the clinic was not assessed in the survey and could not be included in the analysis. Given the higher utilization of traditional health care in Tanzania [12], which goes hand in hand with the utilization of non-prescribed nutritional supplement and herbal drugs during pregnancy [52, 53]. This non-inclusion would bias the women’s’ cost downwards, leading to underestimation of cost. Fourth, in this study, we have assessed the cost of health care utilization and did not take into account whether they were able to afford those costs. If these women or their families had to borrow money or sell properties in order to cover for any of the expenses, that may imply substantial household financial burdens as suggested in previous studies [25, 28, 31]. Lastly, although we reported on just one rural district, we noted that the study setting has many characteristics common to other rural areas in Tanzania; however, further research will be needed to demonstrate generalizability to other settings.

## Conclusion

The vision of free health care for all pregnant women in Tanzania is not a reality to many. Women pay directly and indirectly from their pocket because of a chronic situation of insufficient supplies and drugs. The ongoing country strategic plans that aim at addressing the problem of unavailability of medicine and medical supplies in health facilities are promising [23, 54, 55]. Those strategies should, however, be monitored and evaluated to ensure that even remote facilities are benefiting from these changes.

While the country does work on designing strategies that will allow sustainable availability of healthcare closer to the people, it is paramount not to ignore the high time cost incurred. Addressing limitation in the availability of health care providers in rural settings by implementing the task shifting policy may be plausible. The current country strategy that advocates for the efficient use of community health workers in the health system [56, 57] is an opportunity that may address the shortage of health care workers and help healthcare seekers in rural areas. Yet caution should be taken when delegating clinical assignments to community health workers in order not to jeopardize the quality of care providers.

## Additional files

Additional file 1: Participant Structured Interview Guide and Consent Form. (DOC 142 kb)

## Abbreviations

ANC: Antenatal care
KII: Key informant interview
MHC: Mobile Health Clinic
WHO: World Health Organization

## References

[1] National Bureau of Statistics (2016). Tanzania service provision assessment survey 2014–2015.

[2] DoHPa P, Ministry of Health and Social Welfare (2008). The National Road map Strategic Plan to Accelerate Reduction of maternal, newborn and child deaths in Tanzania.

[3] Plan International (2016). Impact Evaluation of the Mobile Health Clinic.

[4] Neke NM, Gadau G, Wasem J (2018). Policy makers’ perspective on the provision of maternal health services via MHC in Tanzania—findings from key informant interviews. PLoS One.

[5] Ensor T, Ronoh J (2005). Effective financing of maternal health services: a review of the literature. Health Policy.

[6] McIntyre D, Thiede M, Dahlgren G, Whitehead M (2006). What are the economic consequences for households of illness and of paying for health care in low-and middle-income country contexts?. Soc Sci Med.

[7] Perkins M, Brazier E, Themmen E, Bassane B, Diallo D, Mutunga A, Mwakajonga T, Ngobola O (2009). Out-of-pocket costs for facility-based maternity care in three African countries. Health Policy Plan.

[8] Chuma J, Gilson L, Molyneux C (2007). Treatment-seeking behaviour, cost burdens and coping strategies among rural and urban households in coastal Kenya: an equity analysis. Tropical Med Int Health.

[9] World Health Organisation (2016). WHO recommendations on ANC for a positive pregnancy experience.

[10] Kruk ME, Paczkowski M, Mbaruku G, de Pinho H, Galea S (2009). Women’s preferences for place of delivery in rural Tanzania: a population-based discrete choice experiment. Am J Public Health.

[11] Kruk ME, Paczkowski MM, Tegegn A, Tessema F, Hadley C, Asefa M, Galea S (2010). Women’s preferences for obstetric care in rural Ethiopia: a population-based discrete choice experiment in a region with low rates of facility delivery. J Epidemiol Community Health.

[12] National Bureau of Statistics (2016). Tanzania National Demographic and health survey and malaria Indicator survey.

[13] RAaL G, Prime Minister Office-Regional Adminstration and Local Government (2016). Planning and Coordination in Pwani Region.

[14] Drummond MF, Sculpher MJ, Claxton K, Stoddart GL, Torrance GW (2015). Methods for the economic evaluation of health care programmes.

[15] Husereau D, Drummond M, Petrou S, Carswell C, Moher D, Greenberg D, Augustovski F, Briggs AH, Mauskopf J, Loder E (2013). Consolidated health economic evaluation reporting standards (CHEERS)—explanation and elaboration: a report of the ISPOR health economic evaluation publication guidelines good reporting practices task force. Value Health.

[16] Bank of Tanzania (2016). Financial Makerts: Exchange Rates.

[17] Chen YN, Schmitz MM, Serbanescu F, Dynes MM, Maro G, Kramer MR (2017). Geographic access modeling of emergency obstetric and neonatal care in Kigoma region, Tanzania: transportation schemes and programmatic implications. Glob Health Sci Pract.

[18] World Health Organization W, United Nations Population Fund U, United Nations Children’s fund U. Averting maternal death and disability, mailman School of Public Health. In: Monitoring Emergency Obstetric Care: A Handbook. edn. Geneva: World Health Organisation. p. 2009.

[19] Goodman S. A dirty dozen: twelve p-value misconceptions. In: Seminars in hematology: 2008: Elsevier; 2008. p. 135–40. 10.1053/j.seminhematol.2008.04.003 18582619

[20] Fisher RA (1956). Statistical methods and scientific inference.

[21] Neyman J, Pearson ES (1933). IX. On the problem of the most efficient tests of statistical hypotheses. Phil Trans R Soc Lond A.

[22] Sterne JA, Smith GD (2001). Sifting the evidence—what's wrong with significance tests?. Phys Ther.

[23] Ministry of Finance and Planning (2016). National Five year Development Plan 2016/2017-2020/2021.

[24] Gray AM, Clarke PM, Wolstenholme JL, Wordsworth S (2010). Applied methods of cost-effectiveness analysis in healthcare.

[25] Kowalewski M, Mujinja P, Jahn A (2002). Can mothers afford maternal health care costs? User costs of maternity services in rural Tanzania. Afr J Reprod Health.

[26] Budlender D (2008). The statistical evidence on care and non-care work across six countries. Gender and development Programme paper number.

[27] Hardon AP, Akurut D, Comoro C, Ekezie C, Irunde HF, Gerrits T, Kglatwane J, Kinsman J, Kwasa R, Maridadi J (2007). Hunger, waiting time and transport costs: time to confront challenges to ART adherence in Africa. AIDS Care.

[28] Callaghan-Koru JA, McMahon SA, Chebet JJ, Kilewo C, Frumence G, Gupta S, Stevenson R, Lipingu C, Baqui AH, Winch PJ (2016). A qualitative exploration of health workers’ and clients’ perceptions of barriers to completing four ANC visits in Morogoro region, Tanzania. Health Policy Plan.

[29] Stekelenburg J, Kyanamina S, Mukelabai M, Wolffers I, Roosmalen JV (2004). Waiting too long: low use of maternal health services in Kalabo, Zambia. Tropical Med Int Health.

[30] Fogliati P, Straneo M, Brogi C, Fantozzi PL, Salim RM, Msengi HM, Azzimonti G, Putoto G (2015). How can childbirth care for the rural poor be improved? A contribution from spatial modelling in rural Tanzania. PLoS One.

[31] Kruk ME, Hermosilla S, Larson E, Mbaruku GM (2014). Bypassing primary care clinics for childbirth: a cross-sectional study in the Pwani region, United Republic of Tanzania. Bull World Health Organ.

[32] Creswell JW, Clark VLP (2007). Designing and conducting mixed methods research.

[33] James C (1990). Foundations of social theory.

[34] Shaw CR, McKay HD. Juvenile delinquency and urban areas. Chicago; 1942.

[35] Kornhauser RR (1978). Social sources of delinquency: an appraisal of analytic models.

[36] Gage AJ (2007). Barriers to the utilization of maternal health care in rural Mali. Soc Sci Med.

[37] Magadi MA, Agwanda AO, Obare FO (2007). A comparative analysis of the use of maternal health services between teenagers and older mothers in sub-Saharan Africa: evidence from demographic and health surveys (DHS). Soc Sci Med.

[38] Erci B (2003). Barriers to utilization of prenatal care services in Turkey. J Nurs Scholarsh.

[39] McCaw-Binns A, La Grenade J, Ashley D (1995). Under-users of ANC: a comparison of non-attenders and late attenders for ANC, with early attenders. Soc Sci Med.

[40] Kane S, Rial M, Kok M, Matere A, Dieleman M, Broerse JE (2018). Too afraid to go: fears of dignity violations as reasons for non-use of maternal health services in South Sudan. Reprod Health.

[41] Ministry of Health and Social Welfare (2003). National Health Policy.

[42] Penfold S, Shamba D, Hanson C, Jaribu J, Manzi F, Marchant T, Tanner M, Ramsey K, Schellenberg D, Schellenberg JA (2013). Staff experiences of providing maternity services in rural southern Tanzania–a focus on equipment, drug and supply issues. BMC Health Serv Res.

[43] Masters SH, Burstein R, DeCenso B, Moore K, Haakenstad A, Ikilezi G, Achan J, Osei I, Garshong B, Kisia C (2014). Pharmaceutical availability across levels of care: evidence from facility surveys in Ghana, Kenya, and Uganda. PLoS One.

[44] Ngalesoni F, Ruhago G, Robberstad B, Norheim OF (2015). Economic cost of primary prevention of cardiovascular diseases in Tanzania. Health Policy Plan.

[45] National Bureau of Statistics (2015). National Account of Tanzania mainland(2007-2014).

[46] Ensor T, Cooper S (2004). Overcoming barriers to health service access: influencing the demand side. Health Policy Plan.

[47] O'Donnell O (2007). Access to health care in developing countries: breaking down demand side barriers. Cadernos de Saúde Pública.

[48] Udegboka N, Moses J. Reduction of client waiting time through task shifting in northern Nigeria. In: 5th IAS conference on HIV pathogenesis, treatment and prevention, Cape Town. Cape Town: International AIDS Society. p. 2009.

[49] Callaghan M, Ford N, Schneider H (2010). A systematic review of task-shifting for HIV treatment and care in Africa. Human Resource for Health.

[50] Chung J, O’Brien M, Price J, Shumbusho F (2008). Quantification of physician-time saved in a task shifting pilot program in Rwanda. International AIDS Conference.

[51] Leive Adam (2008). Coping with out-of-pocket health payments: empirical evidence from 15 African countries. Bulletin of the World Health Organization.

[52] Marwa KJ, Njalika A, Ruganuza D, Katabalo D, Kamugisha E (2018). Self-medication among pregnant women attending antenatal clinic at Makongoro health Centre in Mwanza, Tanzania: a challenge to health systems. BMC pregnancy and childbirth.

[53] Mohseni M, Azami-Aghdash S, Sheyklo SG, Moosavi A, Nakhaee M, Pournaghi-Azar F, Rezapour A (2018). Prevalence and reasons of self-medication in pregnant women: a systematic review and meta-analysis. Int J Commun Based Nurs Midwif.

[54] Mackintosh M, Tibandebage P, Njeru MK, Kungu JK, Israel C, Mujinja PG (2018). Rethinking health sector procurement as developmental linkages in East Africa. Soc Sci Med.

[55] Wiedenmayer KA, Kapologwe N, Charles J, Chilunda F, Mapunjo S (2015). The reality of task shifting in medicines management-a case study from Tanzania. J Pharm Policy Pract.

[56] Ministry of Health and Social Welfare (2008). Guidelines for implementing the community based health initiatives.

[57] Ministry of Health and Social Welfare (2015). National Community Based Health Policy. Edited by Planning HPa.

