# Supplementary material for: Time and cost associated with utilization of services at mobile health clinics among pregnant women
Source: BMC Health Serv Res. 2018 Dec 3;18:920. doi: 10.1186/s12913-018-3736-z (PMC6276179; doi:10.1186/s12913-018-3736-z)
Supplement: Supplementary file 1 — Participant Structured Interview Guide and Consent Form. (DOC 142 kb) [file 12913_2018_3736_MOESM1_ESM.doc]

**Patient Questionnaire**

**Study of the Costs of Maternal Health Services at the Mobile Health Clinic**

| **Information Sheet and Informed Consent**  I ……………………………………………….. am here on behalf of Institute for Health Management and Research of the University of Duisburg-Essen (Germany) research team. As part of our project, we are working on a research study of the patient costs of accessing maternal health interventions at the mobile vans run by Plan International in Kisarawe District.  **Purpose of the study**  The survey aims to estimate provider and patient’s costs of various maternal health interventions that are provided by the vans like antenatal care and postnatal care so that it could be compared to effectiveness of using this mode of service delivery for maternal health service.  **Participation and what it involves**  The mobile clinic that provides you with maternal health service is the primary focus of this study. We will collect information so that we can calculate what it costs to provide maternal health services at this mobile van. We also want to calculate the costs that patients pay in order to receive these interventions at the mobile health vans. To do this, we will ask you questions about how much time and money you spend getting services that you need at this particular mobile health van. We will also ask you questions about how far away you live and how you live.  **Confidentiality**  The study is anonymous and we will not record your name or any other personal information. Unauthorized persons will have no access to the data collected.  **Risks and Benefits**  We do not expect any harm to happen to you because of participating in this study. There are no direct benefits to you from taking this survey. Your participation will help with planning for the future of maternal health services delivery strategies in this country. This may help design better way to reach women with maternal health interventions.  **Voluntary participation**  Your participation is **voluntary** and you can withdraw from the survey after having agreed to participate. You are free to refuse to answer any question that is asked in the questionnaire. There is no penalty if you do not wish to participate or wish to stop.  **Contact Information**  If you have any questions regarding this survey or the study, please feel free to ask the interviewers on site. You may keep a copy of this information sheet for your records,  If you have any later questions you can contact the principal investigator Nyasule Neke of the National Institute for Medical research, Isamilo Street, P.O.Box 1462…………………….., Tel No…………………….... Mwanza, Tanzania.  **RESPONDENT COPY** |
| --- |

**Patient Questionnaire**

**Study of the Costs of maternal Health services at the mobile health clinic**

| **Information Sheet and Informed Consent**  I ……………………………………………….. am here on behalf of Institute for Health Management and Research of the University of Duisburg-Essen (Germany) research team. As part of our project, we are working on a research study of the patient costs of accessing maternal health interventions at the mobile vans run by Plan International in Kisarawe District.  **Purpose of the study**  The survey aims to estimate provider and patient’s costs of various maternal health interventions that are provided by the vans like antenatal care and postnatal care so that it could be compared to effectiveness of using this mode of service delivery for maternal health service.  **Participation and what it involves**  The mobile clinic that provides you with maternal health service is the primary focus of this study. We will collect information so that we can calculate what it costs to provide maternal health services at this mobile van. We also want to calculate the costs that patients pay in order to receive these interventions at the mobile health vans. To do this, we will ask you questions about how much time and money you spend getting services that you need at this particular mobile health van. We will also ask you questions about how far away you live and how you live.  **Confidentiality**  We will not record your name or any other personal information only the study identification numbers will be used. Unauthorized persons will have no access to the data collected.  **Risks and Benefits**  We do not expect any harm to happen to you because of participating in this study. There are no direct benefits to you from taking this survey. Your participation will help with planning for the future of maternal health services delivery strategies in this country. This may help design better way to reach women with maternal health interventions.  **Voluntary participation**  Your participation is **voluntary** and you can withdraw from the survey after having agreed to participate. You are free to refuse to answer any question that is asked in the questionnaire. There is no penalty if you do not wish to participate or wish to stop.  **Contact Information**  If you have any questions regarding this survey or the study, please feel free to ask the interviewers on site. You may keep a copy of this information sheet for your records,  If you have any later questions you can contact the principal investigator Nyasule Neke of the National Institute for Medical research, Isamilo Street, P.O.Box 1462…………………….., Tel No…………………….... Mwanza, Tanzania.  **Consent to participate**  Signing this consent indicates that you understand what will be expected of you and are willing to participate in this survey.  Participant agrees……………………………….. Participant refuses……………………………………………….  I……………………………………………………………… have read the contents of this form. My questions have been answered. I agree to participate in this study  **Signature of participant**…………………………………………………………………………………………..  **Signature of the research assistant**……………………………………………………………………………..  **Date of signed consent**…………………………………………………………………...................................... |
| --- |

**Patient Questionnaire**

**Study of the Costs of Maternal Health Services at the Mobile health Clinic**

| **Name of the village** | Village code: |
| --- | --- |
| **Name of the nearest Health facility** |  |
| **Parity** |  |
| **Interviewer Name** |  |
| **Date (mm/dd/yyyy)** | / / . **Start time:** |

| **Basic Information/Demographic** | | |  |
| --- | --- | --- | --- |
| NO. | QUESTIONS AND FILTERS | RESPONSES | SKIPS |
| 1 | Enter respondent’s marital status: | Married 0  Single……….1  Cohabiting …2 |  |
| 2 | What is your age? | ………………….Years old |  |
| 3 | During the last 12 months, what was your main activity? | Farming or livestock keeping 0  Fishing 1  Paid Employee (Government) 2  Paid Employee (Private) 3  Self-employed (with employees) 4  Self-employed (without employees) 5  Unpaid helper in family business 6  Not working (available for work) 7  Not working (not available for work) 8  Homemaker/Housewife/ House chores 9  Student 10  Unable to work (old/retired/sick) 11  Other (describe) 12  If Other describe |  |
| 4 | Is this your first time to receive care from this mobile van? | Yes………0  No………..1 | 5 |
| 5 | How many visits have you made to this mobile van in the past six months? | 1 visit 0  2 visits 1  3 visits 2  4 or more visits 3 |  |
| 6 | For which specific maternal health services are you here for today?  (mark all that apply) | Antenatal care 0  Childbirth 1  Postnatal care 2  Others 3  If other describe  If O indicate the number of the visit → |  |

| **Time and Travel Costs** | | | |
| --- | --- | --- | --- |
| NO. | QUESTIONS AND FILTERS | RESPONSES | SKIPS |
| 7 | How did you get here today?  (mark all that apply) | No Yes  Walk 0 1  Bicycle 0 1  Bus or other public transport 0 1  Private car or motorbike 0 1  Other (describe) 0 1  If other specify |  |
| 8 | How much does it normally cost for transport to this place (one way), for yourself and anybody who normally comes with you? | Shillings |  |
| 9 | Do you come to this place and return home on the same day?  If you stay overnight near the point where the mobile health van park, how much does it cost you for accommodation? [only record accommodation required for attending clinic] | Return home on same day 0  Stay overnight 1  If 1 → Shillings |  |
| 10 | How long does it normally take you to get to where the mobile van park to deliver services (one way)? | Hours Mins |  |
| 11 | How long do you normally have to wait at the waiting area before your appointment? | Routine appt…….… Hrs Mins  Emergency appt…... Hrs Mins  Drug refill appt………. Hrs Mins |  |
| 12 | How long does your appointment normally take? [Including all parts of clinic visit, e.g. doctor visit, counseling session, laboratory investigation sessions, pharmacy visit, if applicable] | Routine appt…….… Hrs Mins  Emergency appt…... Hrs Mins  Drug refill appt………. Hrs Mins |  |

| **Costs of Clinic Treatment** | | | |
| --- | --- | --- | --- |
| NO. | QUESTIONS AND FILTERS | RESPONSES | SKIPS |
| 13 | Have you had to pay for any of the following services at this clinic (include any in-kind payments)? | No Yes  Initial registration appt 0 1  Routine appt 0 1  Emergency appt 0 1  Laboratory tests 0 1  X-rays 0 1  Ultrasound………………………. 0 1 |  |
| 14 | [If ‘Yes’ to any of Q13] How much did you pay for each service, and how many times did you pay for this service on your past visits during this pregnancy? | Initial registration apt  Shillings, Times  Routine appt  Shillings, Times  Emergency appt  Shillings, Times  Blood tests  Shillings, Times  X-rays/Ultrasound  Shillings, Times |  |
| 15 | Were there any other services you have had to pay for at this mobile van?  [If YES] Please describe these services, how much you paid for each service, and how many times you paid for this service in the past 6 months. | Other 1:  Shillings, Times  Other 2:  Shillings, Times  Other 3:  Shillings, Times |  |
| 16 | Have you had to pay for any drugs or other health supplies that were needed for your maternal care, or have you ever been given a prescription to go buy the drugs or other health supplies?  [If YES] How much have you spent on these drugs or health supplies in the last 6 months? | No 0  Yes 1  If 1 → Shillings Shillings |  |
| 17 | Have you made any informal payments to staff at this health van in order to receive treatment?  [If YES] How much have you paid in the last 6 months (include value of in-kind payments)? | No 0  Yes 1  If 1 → Shillings Shillings |  |
| 18 | Apart from the things we have discussed, are there any other costs you have had to pay in order to get services at this mobile van? (for example childcare, lost wages)  [If YES] Please describe what the costs were for, how much you paid, and how many times you had to pay this in the last 6 months. | No 0  Yes 1  Other 1:  Shillings, Times  Other 2:  Shillings, Times  Other 3:  Shillings, Times |  |

| **Costs as a Barrier to Care** | | | |
| --- | --- | --- | --- |
| NO. | QUESTIONS AND FILTERS | RESPONSES | SKIPS |
| 19 | Have you ever missed a scheduled clinic appointment because you could not afford to pay for transport, childcare, or other costs? | No 0  Yes 1 |  |
| 20 | If YES how often? | Very often 0  Often 1  Occasionally 2 |  |
| 21 | Have you ever wanted to come to the clinic for an unscheduled visit but could not come because of the cost? | No 0  Yes 1 |  |
| 22 | If YES how often? | Very often 0  Often 1  Occasionally 2 |  |

| **Time as a Barrier to Care** | | | |
| --- | --- | --- | --- |
| NO. | QUESTIONS AND FILTERS | RESPONSES | SKIPS |
| 23 | Have you ever missed a scheduled clinic appointment because you did not have time? | No 0  Yes 1 |  |
| 24 | If YES how often? | Once 0  Twice 1  More than twice 2 |  |
| 25 | Have you ever wanted to come to the clinic for an unscheduled visit but could not come because of lack of time? | No 0  Yes 1 |  |
| 26 | If YES how often? | Once 0  Twice 1  More than twice 2 |  |

| ***Cost to the Caregivers** | | | | | |
| --- | --- | --- | --- | --- | --- |
| NO. | QUESTIONS AND FILTERS | RESPONSES | | | SKIPS |
| 27 | Does anyone take care of you because of your **current condition**? If so who does that | Immediate f\member 0  Distant relative 1  Maid\paid worker 2  Friend 3  Neighbor……………………….. .. 4 | | |  |
| 28 | How long do they normally use taking care of you in a normal day? | Hours …………………… | | |  |
| 29 | Have you make any payments to these caregivers?  [If YES] How much have you paid in the last 6 months (include value of in-kind payments)? | No 0  Yes 1  If 1 → Shillings | | |  |
| 30 | Any suggestions about the cost of maternal health services? | | | | |
|  | | | **End Time:** |  | |

| ***Interviewer Comments:*** |
| --- |

*“Thank you for taking part in this study.”*
